# Diverse Mobile Genetic Elements and Conjugal Transferability of Sulfonamide Resistance Genes (*sul1*, *sul2*, and *sul3*) in *Escherichia coli* Isolates From *Penaeus vannamei* and Pork From Large Markets in Zhejiang, China

**DOI:** 10.3389/fmicb.2019.01787

**Published:** 2019-08-02

**Authors:** Han Jiang, Hui Cheng, Yi Liang, Shengtao Yu, Ting Yu, Jiehong Fang, Cheng Zhu

**Affiliations:** Key Laboratory of Marine Food Quality and Hazard Controlling Technology of Zhejiang Province, College of Life Sciences, China Jiliang University, Hangzhou, China

**Keywords:** sulfonamide resistance genes, *Escherichia coli*, mobile genetic elements, insertion sequences, conjugation

## Abstract

High prevalence rates of sulfonamide resistance genes *sul1*, *sul2*, and *sul3* have been observed in Gram-negative bacteria isolated from humans, domestic animals, and aquaculture species worldwide. We investigated the distribution characteristics, location, conjugative transferability, and genetic environments of *sul* genes from *Escherichia coli* isolates collected from *Penaeus vannamei* and pork samples from three large markets in Zhejiang, China. The prevalence rates of *sul* genes in sulfonamide-resistant *E. coli* isolates from *P. vannamei* and pork samples were 90.0 and 88.6%, respectively, and the prevalence of *sul1* and *sul2* was significantly higher than that of *sul3* (*p* < 0.05). Twenty-four representative *sul*-positive *E. coli* isolates were analyzed in detail. Southern blot hybridization confirmed that *sul* genes of *E. coli* isolates were located on plasmids and/or chromosomes. Transfer of resistance through conjugation was observed in all 18 *E. coli* isolates harboring *sul* genes on plasmids. Replicon typing identified seven different incompatibility groups and IncF was the dominant replicon type among *sul* gene-containing plasmids from both sources. PCR walking analysis indicated that 87.5% (35/40) of *sul* gene-related fragments carried insertion sequences (ISs) belonging to a variety of families in diverse sites, with IS*26* occurring most frequently. In addition, the *sul1* gene was detected mainly in fragments carrying class 1 integrons. Co-location on the same fragment with resistance genes that may contribute to the persistence and dissemination of *sul1* and/or *sul2* genes. The diversity of mobile genetic elements and resistance genes adjacent to *sul3* was much lower than those adjacent to *sul1* and *sul2*, especially those located in chromosomes, which reduced the transmission potential of the *sul3* gene. In conclusion, combined with the results of clonal relatedness analysis by PFGE and MLST of 24 representative *E. coli* isolates from *P. vannamei* and pork samples, it showed that a small number of *sul* genes were vertically transmitted among *E. coli* from *P. vannamei* and that horizontal gene transfer was likely the main transmission mechanism of *sul* genes from both sources. Our results provide important information to better understand the risk of transmission of *sul* genes from seafood and meat to humans.

## Introduction

Sulfonamides, which are fully synthetic antibiotics, have been widely used to treat bacterial and protozoan infections in humans, domestic animals, and aquaculture species (Blahna et al., 2006; Peixoto et al., 2016). Although the use of sulfonamides in human medicine has decreased in developed countries, they are still frequently used in developing Asian countries owing to their low price and availability (Changkaew et al., 2014). In veterinary medicine, sulfonamides are considered “high priority” due to high usage and their high potential to enter the environment (Heuer and Smalla, 2007; Wang et al., 2014).

In the past decade, high prevalence rates of sulfonamide resistance have been observed in mainly Gram-negative bacteria isolated from animals and humans all over the world (Wu et al., 2010; Card et al., 2016; Ben et al., 2017). Resistance to sulfonamides occurs principally through the acquisition of the alternative dihydropteroate synthase (DHPS) gene *sul*, the product of which has a low affinity for sulfonamides (Changkaew et al., 2014). Unlike resistance genes to other classes of antimicrobials such as tetracycline, which are encoded by many different genes, only three known sulfonamide resistance (*sul*) genes (*sul1*, *sul2*, and *sul3*) have been identified (Wu et al., 2010; Ben et al., 2017). Based on alignments using MegAlign software, the nucleotide sequences of *sul1*, *sul2*, and *sul3* are approximately 50% similar (Hsu et al., 2014).

Horizontal or vertical transfer of mobile genetic elements (MGEs) that harbor resistance genes is considered the main mechanism of dissemination of antibiotic resistance genes (Rehman et al., 2017). *sul* genes have been identified on both chromosomes and plasmids and are often associated with MGEs such as transposons, integrons and insertion sequences (ISs) (Wu et al., 2010). MGEs enable translocation of *sul* genes between chromosomes and plasmids. In addition, plasmids carrying *sul* genes can spread among bacteria of the same or different species or genera by conjugation or transformation, thereby disseminating *sul* genes.

*Penaeus vannamei*, also known as Pacific whiteleg shrimp, are among the most popular culturable penaeid shrimp in China, and China has been the world’s largest producer of *P. vannamei* since 2001 (Global Aquaculture Production 1950−2016, FAO)^1^. Similarly, in China, pork accounts for approximately 64% of all meat production and is consistently the most consumed meat (Ministry of Agriculture and Rural Affairs of the People’s Republic of China, March 2019)^2^. Moreover, sulfonamides are among the most commonly used antibiotics at swine farms, second only to tetracyclines (Hsu et al., 2014), and are widely used in *P. vannamei* culture for the prevention and treatment of common bacterial diseases (Gao et al., 2012). Excessive usage of sulfonamides in the shrimp farming and pig breeding industries in China imposes widespread selective pressures on bacteria, leading to the enrichment of sulfonamide resistant strains in shrimp and pork products that are capable of spreading between different environments (Liu et al., 2019; Yuan et al., 2019). In our study, *P. vannamei* and pork were chosen to study the prevalence of sulfonamide resistance in three large markets in Zhejiang, China to assess (i) whether differences occur in the distribution of *sul* genes in *Escherichia coli* isolates from samples of two different host species collected from markets, (ii) whether vertical or horizontal transmission of sulfonamide resistant *E. coli* occurs between *P. vannamei* and pork in market environments, despite the different growth conditions of the two animals and (iii) whether regional spreading of sulfonamide resistant *E. coli* in the same or different host species occurs between different markets.

*Escherichia coli* are ubiquitous commensal bacteria, and certain strains are zoonotic pathogens (Zhang et al., 2013). *E. coli* are also considered effective model organisms for studying the essential processes of life due to their fast growth rate, minimal nutritional requirements, comprehensively understood biological processes, and extensively characterized genetics (Jiang et al., 2016; Paraoan et al., 2017). More importantly, *E. coli* are often used to monitor antimicrobial resistance, as they have a wide range of hosts and can easily acquire resistance genes via horizontal gene transfer (Fang et al., 2019). Many nutritious aquatic and meat products are considered reservoirs of *E. coli* as well as some antimicrobial resistance genes (Alvarez-Fernandez et al., 2013; Skočková et al., 2015; Boss et al., 2016).

Here we investigated *sul* genes in *E. coli* isolates collected from *P. vannamei* and pork samples from three large markets in Zhejiang Province, China. The clonal relatedness among the representative *sul*-positive *E. coli* isolates was analyzed by pulsed-field gel electrophoresis (PFGE) and multilocus sequence typing (MLST). In addition, the location, genetic environment, and conjugative transferability of *sul* genes were further researched to assess the potential transmission ability of *sul* genes in *E. coli* from two different animal host species.

## Materials and Methods

### Shrimp and Pork Sampling and *E. coli* Isolation

A total of 180 *P. vannamei* and 180 pork samples were collected from three different open markets (*n* = 60 per market) in Zhejiang Province, China between June 2017 and June 2018 (Table 1). Samples were collected from each market every 4 months (June 2017, October 2017, February 2018, and June 2018). A total of 90 samples were collected from the three markets at each visit, including 15 *P. vannamei* and 15 pork samples from each market. The methods for sample treatment and *E. coli* isolation and identification are described in detail in our previously published studies (Cheng et al., 2019; Fang et al., 2019). Following the previous protocol, one *E. coli* isolate was obtained from each sample (Changkaew et al., 2014; Boss et al., 2016; Cheng et al., 2019; Fang et al., 2019). Colonies were stored at −80°C until further examination.

**TABLE 1:** Distribution characteristics of sulfonamide resistance (*sul*) genes present in *Escherichia coli* isolated from *Penaeus vanmamei* and pork samples from three different open markets.

		**Total no. of samples**	**No. of *E. coli* isolates** ***n* (%)**	**Sulfisoxazole resistance** ***n* (%)**	**Individual *sul1-*positive** ***n* (%)**	**Individual *sul2-*positive** ***n* (%)**	**Individual *sul3-*positive** ***n* (%)**	***sul1* + *sul2-*positive** ***n* (%)**	***sul1* + *sul3-*positive** ***n* (%)**	***sul2* + *sul3-*positive** ***n* (%)**	***sul1* + *sul2* + *sul3-*positive** ***n* (%)**	***suls*-negative** ***n* (%)**
*P. vanmamei*	Market-1	60	50(83.3)	21(42.0)	5(23.8)	4(19.0)	1(4.8)	5(23.8)	1(4.8)	2(9.5)	0 (0)	3(14.3)
	Market-2	60	48(80.0)	14(29.2)	3(21.4)	3(21.4)	0(0)	5(35.7)	2(14.3)	1(7.1)	0 (0)	0(0)
	Market-3	60	42(70.0)	15(35.7)	3(20.0)	3(20.0)	1(6.7)	4(26.7)	1(6.7)	1(6.7)	0 (0)	2(13.3)
	Total	180	140(77.7)	50(35.7)	11(22.0)	10(20.0)	2(4.0)	14(28.0)	4(8.0)	4(8.0)	0 (0)	5(10.0)
Pork	Market-1	60	60(100.0)	31(62.0)	3(9.7)	7(22.6)	1(3.2)	10(32.3)	3(9.7)	3(9.7)	0 (0)	4(12.9)
	Market-2	60	60(100.0)	45(75.0)	17(37.8)	10(22.2)	3(6.7)	10(22.2)	0(0)	3(6.7)	0 (0)	2(4.4)
	Market-3	60	60(100.0)	29(58.0)	13(44.8)	7(24.1)	0(0)	3(10.3)	0(0)	0(0)	0 (0)	6(20.7)
Total		180	180(100.0)	105(58.3)	33(31.4)	24(22.9)	4(3.8)	23(21.9)	3(2.9)	6(5.7)	0 (0)	12(11.4)

### Phenotypic Screening for Sulfonamide Resistance

All confirmed *E. coli* isolates collected from *P. vannamei* and pork samples were assessed for susceptibility to sulfisoxazole using the disk diffusion method on Mueller-Hinton (MH) agar (Oxoid Ltd., Basingstoke, United Kingdom) in accordance with the guidelines of the (Clinical and Laboratory Standards Institute [CLSI], 2017). According to CLSI guidelines, sulfisoxazole can be used to test all known types of sulfonamide resistance. Sulfisoxazole (300 μg) antibiotic disks were obtained from Hangzhou Microbial Reagent Co., Ltd. (Hangzhou, China). *E. coli* isolates were incubated overnight at 37°C and diluted to a turbidity equivalent to a 0.5 McFarland standard. The inoculated MH agar plates were then incubated at 37°C for 24 h, and the size of the zone of inhibition was used to classify isolates as susceptible, intermediate or resistant according to CLSI guidelines. *E. coli* ATCC 25922 was used as a sensitivity control.

### Detection of *sul* Genes

Polymerase chain reaction (PCR) was used to detect the presence of *sul* genes in all sulfisoxazole-resistant *E. coli* isolates. The boiling method described in our previous studies was used to obtain genomic DNA templates (Cheng et al., 2019). The primers used to detect *sul* genes are shown in Table 2. Each PCR amplification reaction was performed in a 25 μL mixture containing 400 nM each primer, 10 × PCR buffer, 200 mM each deoxynucleotide triphosphate (dNTP), 5 U Ex-Taq DNA polymerase (Takara-Bio, Beijing, China) and 250 ng DNA as the template. PCR amplification was initiated by incubating the reaction mixture at 94°C for 1 min; followed by 30 cycles at 98°C for 30 s, annealing at 56°C (*sul2*) or 55°C (*sul1* and *sul3*) for 30 s, and extension at 72°C for 30 s; and a final extension at 72°C for 10 min. PCR products (5 μL) were mixed with 1 μL 6 × loading buffer dye and analyzed by electrophoresis on a 1.2% agarose gel. Positive and negative controls were included in all PCR reactions.

**TABLE 2:** Primers and PCR amplification conditions for detection of *sul1*, *sul2*, and *sul3* genes.

**Target**	**Primer^a^**	**Sequence (5′-3′)**	**Amplicon size (bp)**
*sul1*	sul1F	GGCCGATGAGATCAGACGTA	413
	sul1R	TTTGAAGGTTCGACAGCACG	
*sul2*	sul2F	GCAGGCGCGTAAGCTGA	657
	sul2R	GGCTCGTGTGTGCGGATG	
*sul3*	sul3F	ATTGATTTGGGAGCCGCTTC	412
	sul3R	AAAAGAAGCCCATACCCGGA	

*^*a*^Primers used in this study for the detection of *sul* genes were designed using Primer Premier 5.0 software according to primer design principles.*

### Clonal Analysis by Molecular Typing

Twelve *sul*-positive *E. coli* isolates from *P. vannamei* and 12 *sul*-positive *E. coli* isolates from pork samples were selected from all *sul*-positive *E. coli* isolates and further analyzed by *Xba*I-PFGE typing. The selection criteria for these 24 strains were based on the distribution characteristics and isolation rates of each sulfonamide resistance gene and included all sulfonamide resistance gene arrays (for a detailed description see Table 3). PFGE patterns were compared using BioNumerics Version 7.6 (Applied Maths, Inc., Austin, TX, United States) with a similarity cutoff of 90% to indicate identical groups. The same isolates were also analyzed by MLST using seven housekeeping genes (*adk*, *fumC*, *gyrB*, *icd*, *mdh*, *purA*, and *recA*) and the primers and protocol specified in the online MLST database for *E. coli*^3^.

**TABLE 3:** Characteristics of 24 *sul*-positive *Escherichia coli* isolates.

**Isolate^a^**	**Source**	***suls* genes harboring**	**Location of *suls* genes**	**Plasmid replicon types^b^**	**Transfer by conjugation of plasmids**
1PV-15	*P. vanmamei*	*sul1*	*sul1*: plasmid	F, FIA, Y, K	+
2PV-60	*P. vanmamei*	*sul1*	*sul1*: plasmid	F, FIB, K	+
3PV-02	*P. vanmamei*	*sul1*	*sul1*: chromosome	/	/
1PV-01	*P. vanmamei*	*sul2*	*sul2*: plasmid	F	+
2PV-19	*P. vanmamei*	*sul2*	*sul2*: plasmid	F, K	+
3PV-52	*P. vanmamei*	*sul2*	*sul2*: plasmid	F	+
1PV-44	*P. vanmamei*	*sul3*	*sul3*: plasmid	F, FIB, K	+
1PV-38	*P. vanmamei*	*sul1* + *sul2*	*sul1*: plasmid *sul2*: plasmid	F, K	+
2PV-22	*P. vanmamei*	*sul1* + *sul2*	*sul1*: plasmid *sul2*: plasmid	F	+
3PV-15	*P. vanmamei*	*sul1* + *sul2*	*sul1*: chromosome *sul2*: chromosome	/	/
2PV-02	*P. vanmamei*	*sul1* + *sul3*	*sul1*: plasmid and chromosome *sul3*: chromosome	F, K	+
3PV-24	*P. vanmamei*	*sul2* + *sul3*	*sul2*: plasmid *sul3*: chromosome	F, FIB, K	+
1PO-01	Pork	*sul1*	*sul1*: both on plasmid and chromosome	F, FIA, K	+
2PO-57	Pork	*sul1*	*sul1*: both on plasmid and chromosome	F, FIA, K	+
3PO-15	Pork	*sul1*	*sul1*: both on plasmid and chromosome	F, FIB, K	+
1PO-29	Pork	*sul2*	*sul2*: plasmid	F, FIB, K, I1	+
2PO-14	Pork	*sul2*	*sul2*: plasmid	F, K	+
3PO-48	Pork	*sul2*	*sul2*: both on plasmid and chromosome	F, K	+
2PO-11	Pork	*sul3*	*sul3*: chromosome	/	/
1PO-20	Pork	*sul1* + *sul2*	*sul1*: plasmid *sul2*: plasmid	F, FIB, K	+
2PO-36	Pork	*sul1* + *sul2*	*sul1*: plasmid *sul2*: plasmid	F, I1, N, K	+
3PO-14	Pork	*sul1* + *sul2*	*sul1*: chromosome *sul2*: chromosome	/	/
1PO-27	Pork	*sul1* + *sul3*	*sul1*: chromosome *sul3*: chromosome	/	/
1PO-58	Pork	*sul2* + *sul3*	*sul2*: chromosome *sul3*: chromosome	/	/

*^*a*^The first number of each isolate name indicates the market from which it was collected (market 1, 2, or 3). PV indicates that the source of the isolate was *Penaeus vannamei*, whereas PO indicates that the source of the isolate was pork. The last two numbers indicate the serial number of the isolate. ^*b*^Plasmid replicon types were determined using the method of Carattoli et al. (2005).*

### Location and Transferability of *sul-*Genes

The locations of *sul* genes were determined by Southern blotting with digoxigenin (DIG)-labelled *sul1*, *sul2*, and *sul3* probes. Plasmid and genomic DNA were extracted using an EndoFree Plasmid Maxi Kit (Qiagen, Germantown, MD, United States) and a Bacteria Genomic DNA Extraction Kit (Biotek, Beijing, China), respectively. Plasmid and genomic DNA were digested with *Bam*HI (Fermentas, Waltham, MA, United States) at 37°C for 5 h and then used for Southern blotting. Southern blotting, including DIG-DNA labeling, determination of labeling efficiency, DNA transfer, DNA fixation, hybridization and immunological detection, was performed using a DIG High Prime DNA Labeling and Detection Starter Kit II (Roche Applied Sciences, Penzberg, Germany).

Conjugation assays were performed to investigate the transferability of *sul* genes. The 18 *E. coli* isolates that were found by location analysis to harbor *sul* genes in plasmids were used as donor strains. The rifampin-resistant *E. coli* strain NK5449 was used as the recipient strain. The donor strain and the recipient strain were mixed at a ratio of 1:4 (v/v) and incubated overnight at 37°C in LB broth. Transconjugants were selected on LB agar plates containing 1 mg/mL sulfisoxazole and 1 mg/mL rifampicin. Plasmid DNA of transconjugants was extracted and the presence of *sul* genes was confirmed by PCR analysis.

### Replicon Typing of Plasmids

The plasmid replicon types of all 18 transconjugants mentioned above were determined using the previously described PCR-based replicon typing (PBRT) method targeting 18 replicon types (Carattoli et al., 2005). Plasmids of all 18 transconjugants harboring *sul* genes were confirmed by Southern blotting using *sul* gene probes.

### Genetic Context Analysis of *sul-*Genes

The genetic context of the *sul* genes of the 24 *sul*-positive *E. coli* isolates mentioned above was assessed using the PCR walking method with a Universal GenomeWalker^TM^ 2.0 Kit (Clontech, Mountain View, CA, United States) and the primers listed in Table 4. The upstream adaptor primer AP-F and the downstream *sul* gene primers sul1R-1, sul2R-1, and sul3R-1 targeted the upstream genetic context of the *sul1*, *sul2*, and *sul3* genes, respectively. The downstream adaptor primer AP-R and the upstream *sul* gene primers sul1F-1, sul2F-1, and sul3F-1 targeted the downstream genetic context of the *sul1*, *sul2*, and *sul3* genes, respectively. PCR products were purified using an Axyprep DNA Gel Extraction Kit (Axygen, Hangzhou, China) and sequenced by Sangon Biotech Co., Ltd. (Shanghai, China). Annotation analysis of the nucleotide sequences was performed using the BLAST program^4^ and ISs were analyzed using the ISfinder program^5^. Schematic diagrams of the genetic contexts of *sul* genes were drawn using Inkscape software Version 0.92.3.

**TABLE 4:** Primers for PCR amplification of flanking sequences

**Primers**	**Sequences (5′-3′)**
AP-F	CCCTCTAGATGCATGCTCGAGACTATAGGGCACGCGTGGT
AP-R	TTGGTACCGAGCTCGGATCCACTATAGGGCACGCGTGGT
sul1F-1	CCCTCTAGATGCATGCTCGAGGCGCTGACTACGTCCGCACCCA
sul1R-1	TTGGTACCGAGCTCGGATCCGTCTGATCCGACTCGCAGCATTTCG
sul2F-1	CCCTCTAGATGCATGCTCGAGTCGATTTGCCGGTGCTTCTGTCTG
sul2R-1	TTGGTACCGAGCTCGGATCCATGCCGGACCGAGGTCGATCACAT
sul3F-1	CCCTCTAGATGCATGCTCGAGGTGAAATCTCGTTTAGCACCA ACTCTTGCA
sul3R-1	TTGGTACCGAGCTCGGATCCTCAATCACATCTGCTCCATCT TCAACCA

## Results and Discussion

### *sul* Gene Distribution in *E. coli*

As shown in Table 1, 35.7% (50/180) of the *E. coli* isolates collected from *P. vannamei* samples were sulfisoxazole resistant, and 58.3% (105/180) of the *E. coli* isolates collected from pork samples were sulfisoxazole resistant. *E. coli* isolates from pork and aquatic food products often display resistance against sulfonamide, as this antibiotic has been widely used to treat bacterial and protozoan infections in swine and aquaculture. Even in the absence of selective pressure, sulfonamide-resistant bacteria can remain stable in the environment for 5–10 years and persist longer than sulfonamide itself (Gao et al., 2012).

As shown in Supplementary Figure S1, *sul1*, *sul2*, and *sul3* were detected by PCR. The distribution characteristics of *E. coli sul* genes were similar between the two sources. Among all 50 sulfisoxazole-resistant *E. coli* isolates from *P. vannamei* samples, 90.0% (45/50) harbored *sul* genes, and among all 105 sulfisoxazole-resistant *E. coli* isolates from pork samples, 88.6% (93/105) harbored *sul* genes. The prevalence of *sul* genes was nearly as high as the rate of sulfisoxazole resistance in *E. coli* isolates. The remainder of sulfisoxazole resistance in *sul*-negative isolates was likely due to mechanisms such as mutations in the chromosomal DHPS gene *flop* (Changkaew et al., 2014).

The prevalence rates of individual *sul1* and *sul2* genes in *E. coli*, although variable among sampling sites, were consistently higher than that of individual *sul3* gene (*p* < 0.05). Moreover, the prevalence of combined *sul1* and *sul2* genes was significantly higher than that of other gene combinations at each sampling point. This is consistent with trends previously reported in the literature (Antunes et al., 2005; Hammerum et al., 2006; Byrne-Bailey et al., 2008; Hoa et al., 2008; Gao et al., 2012; Lopes et al., 2015; Ma̧ka et al., 2015), although other studies have reported higher prevalence of *sul3* in *Salmonella* and *E. coli* isolates from pigs and poultry sources in Canada (Kozak et al., 2009) and Thailand (Chuanchuen and Padungtod, 2009). These two studies also reported isolates carrying *sul1*, *sul2*, and *sul3* genes, a combination that was not detected in our study. This discrepancy may be due to differences in the transmission ability of the three genes in the *E. coli* isolates we selected, a possibility that is discussed in the following sections.

### Clonal Relatedness of *sul*-Positive *E. coli* Strains

**FIGURE 1:**
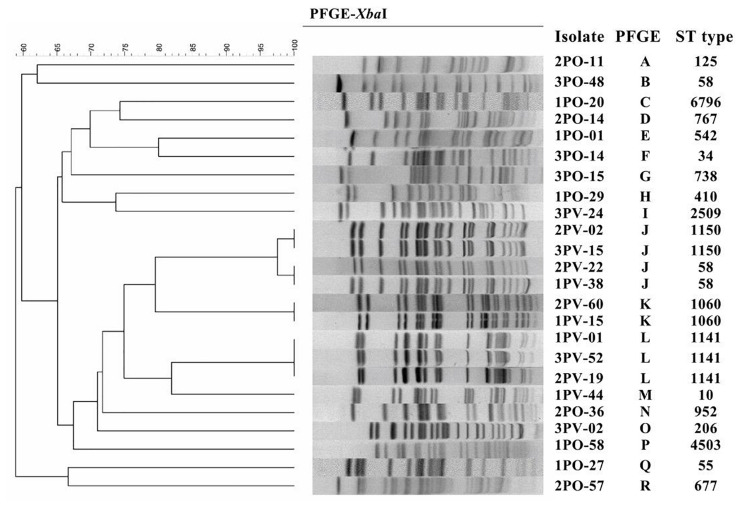
Pulsed-field gel electrophoresis (PFGE) and multilocus sequence typing (MLST) of 12 *sul*-positive *Escherichia coli* isolates from *Penaeus vannamei* and 12 *sul*-positive *E. coli* isolates from pork products.

Among the 24 *sul*-positive *E. coli* isolates, PFGE analysis identified 19 pulsotypes that could be grouped into 18 groups. In addition, MLST analysis identified 19 distinct sequence types (STs). The results of PFGE and MLST analyses are summarized in Figure 1.

PFGE results showed that, of the 12 strains detected in *P. vannamei* samples, 2PV-02 and 3PV-15 had 100% similar fingerprint patterns, 2PV-22 and 1PV-38 had 100% similar fingerprint patterns, 2PV-60 and 1PV-15 had 100% similar fingerprint patterns, and 1PV-01, 3PV-52, and 2PV-19 had 100% similar fingerprint patterns. The remaining three strains (3PV-24, 1PV-44, and 3PV-02) each had unique pulsotypes. These results suggest potential clonal dissemination of *sul*-positive *E. coli* isolates from *P. vannamei* samples. Although the *E. coli* isolates in this study were recovered from three different large open markets on different sampling days, all *P. vannamei* at these markets were reared in the same large-scale freshwater shrimp farm in Zhejiang Province. Therefore, high clonality of *E. coli* strains circulating on the shrimp farm was likely. Additionally, similarities between *E. coli* strains isolated from different large open markets indicates regional spreading of clones, which may have occurred by several possible pathways, including human contact, water sources, animals and fomites (Alves et al., 2018). On contrary, all 12 strains collected from pork samples had different pulsotypes, demonstrating wide genomic diversity. The possible non-clonal dissemination of *sul* genes in *E. coli* isolates from pork samples might be effected by MGEs via horizontal gene transfer (HGT) (Zhao et al., 2018). However, further genomic analysis is necessary to confirm this hypothesis.

Based on the results of MLST combined with the results of PFGE, the four group J strains belonged to two different STs, while the remaining MLST and PFGE patterns were highly correlated. Moreover, different pulsotypes and STs were identified in isolates from *P. vannamei* samples and pork samples. These differences might be related to the different regions where the aquatic and pork products from each large market originated, different conditions at each farm and various transportation routes of the two types of products (Alves et al., 2018).

### Localization of *sul* Genes and Characterization of *sul* Gene-Harboring Plasmids

Southern blot assays showing the locations of *sul1*, *sul2*, and *sul3* are shown in Supplementary Figure S2. Location of *sul* genes in the 24 *E. coli* strains is summarized in Table 3. Among the 12 *E. coli* strains detected in *P. vannamei* samples, eight strains carried *sul* genes only on plasmids, two strains carried *sul* genes only on chromosomes and two strains carried *sul* genes on both plasmids and chromosomes. Among the 12 *E. coli* strains isolated from pork samples, four strains carried *sul* genes only on plasmids, four strains carried *sul* genes only on chromosomes and four strains carried *sul* genes on both plasmids and chromosomes.

Several researchers have highlighted the vital role of plasmids in the carriage and dissemination of *sul* genes (Wu et al., 2010; Ma̧ka et al., 2015). In our study, 18 *E. coli* isolates harboring *sul* genes on plasmids were selected for conjugation assays, with a conjugation rate of 100% (Table 3). Replicon typing identified seven different incompatibility groups (F, FIA, FIB, Y, K, I1, and N) for the plasmids (Table 3). Overall, IncF (carrying F alone or in combination with FIA or/and FIB as a multireplicon) seemed to be the most common replicon associated with *sul* genes among *E. coli* isolates from both *P. vannamei* and pork samples. IncF plasmids are thought to have a narrow host range and to be conjugative. IncF plasmids seem to be well adapted to *E. coli*, as they are found in more than 50% of *E. coli* strains from different sources (Wu et al., 2010; Hayashi et al., 2018). Of the 24 *E. coli* isolates in this study, 18 were found to harbor IncF plasmids. Therefore, *sul* genes located on IncF plasmids may be disseminated in *E. coli* by conjugation.

Of the two plasmids from *E. coli* isolates from *P. vannamei* that carried both *sul1* and *sul2* genes, one harbored the F replicon alone and the other harbored an F-K multireplicon. Of the two plasmids from *E. coli* isolates from pork samples, one harbored an F-FIB-K multireplicon and the other harbored an F-I1-N-K multireplicon. The I1 and N replicons were found only in *E. coli* isolates from pork samples. However, due to the limited number of strains obtained from each source, testing of additional strains is required to determine whether specific replicon profiles are associated with *P. vannamei* or pork.

Overall, no clear association was identified between replicon types and specific *sul* genes and/or sample sources. However, the localization of *sul* genes on wide-spread conjugative replicons such as IncF and other multireplicons very likely contributes to the dissemination of sulfonamide resistance (Wu et al., 2010).

### Genetic Environments of *sul* Genes

**FIGURE 2:**
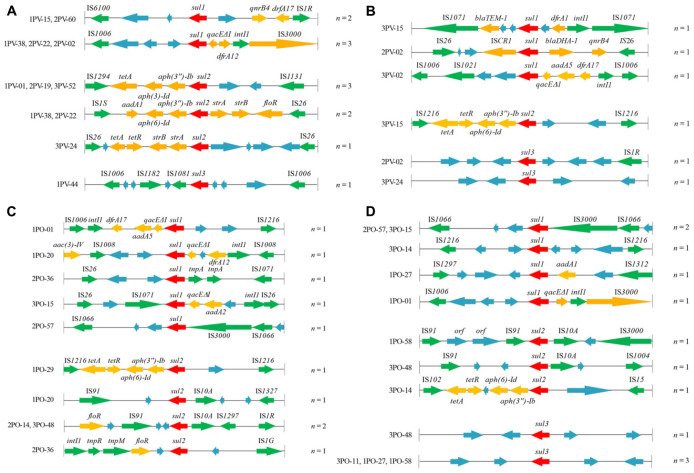
Genetic organization of *sul* gene-associated regions in **(A)** plasmids of 12 *sul*-positive *Escherichia coli* isolates from *Penaeus vannamei*; **(B)** chromosomes of 12 *sul*-positive *E. coli* isolates from *P. vannamei*; **(C)** plasmids of 12 *sul*-positive *E. coli* isolates from pork products; and **(D)** chromosomes of 12 *sul*-positive *E. coli* isolates from pork products presented with their isolate numbers. The orientation of each gene and insertion element is indicated by arrows. The same units are shown in the same color. The same functional units or unknown functional units are shown in the same color (red, *sul* genes; yellow, antibiotic resistance genes other than *sul* genes; green, mobile genetic elements; blue, unknown functional unit). Names of sequence units are indicated above or below the arrows, and sequence units with unknown functions have been left blank.

In this study, we analyzed the structural characteristics of the regions approximately 5 kb upstream and 5 kb downstream of *sul* genes in 24 *E. coli* isolates using the PCR walking method. Representative primary PCR products are shown in Supplementary Figure S3. The genetic environments of *sul1*, *sul2*, and *sul3* genes in 24 *E. coli* isolates were shown in Figure 2.

ISs are the simplest autonomous MGEs capable of altering the expression of neighboring genes (Zhao et al., 2018). In the present study, 87.5% (35/40) of *sul* gene-related fragments were found to carry ISs belonging to a variety of families in diverse sites. All were intact with their inverted repeats (IR_R_ and IR_L_) still identifiable. Among these, the IS*6* family element IS*26*, which has been reported to play a vital role in the dissemination of resistance determinants in Gram-negative bacteria (Partridge et al., 2018), was the most frequently occurring IS among the 24 *E. coli* strains. Moreover, *sul2* genes on both plasmids (1PO-20, 2PO-14, and 3PO-48) and chromosomes (1PO-58 and 3PO-48) of *E. coli* isolates from pork samples carried IS*91* and IS*10A*, which suggested that IS*91*-*sul2*-IS*10A* may mediate the dissemination of *sul2* genes in *E. coli* in pork. Other IS families found in our study, such as IS*6100*, IS*IR*, and IS*1006*, may also have the ability to transfer sulfonamide resistance genes by “cut-and-paste” or “copy-out-paste-in” mechanisms (Partridge et al., 2018). In addition, IS common regions (IS*CRs*) are complex genetic elements that can integrate non-genetic cassette antibiotic resistance genes (Cheng et al., 2019). IS*CR1* elements located on the chromosome of the 2PV-02 isolate from *P. vannamei* were closely linked to the *sul1* gene. IS*CR1* appears to be responsible for capturing and transferring many different antibiotic resistance genes and is often found adjacent to the *ori* end of IS*CR1* in complex class 1 integrons (Toleman et al., 2006; Partridge et al., 2018). However, in the present study, IS*CR1* was located downstream of the *sul1* gene; therefore, its potential role in the *sul1* gene transfer requires further study. Moreover, researchers have reported that IS*CR2* is associated with several different resistance genes, in particular *sul2* in the Gl*sul2* genomic island (Partridge et al., 2018). Thus, more genetic environments of *sul* genes must be analyzed to confirm the relationship between *sul* genes and IS*CR* elements.

In the present study, the *sul1* gene was detected mostly in fragments carrying the *intI1* gene of class 1 integrons (9/18), which are recognized as playing an essential role in the dissemination of multiple antimicrobial resistance genes (Lopes et al., 2015). Class 1 integrons are MGEs and usually carry the *sul1* gene in their 3’ conserved region along with *qac*Δ*E1*, which encodes a semifunctional derivate of the quaternary ammonium compounds resistance gene *qacE* (Sandvang et al., 2002). *aadA* and *drf* genes are also prevalent in class 1 integrons adjacent to *qac*Δ*E1* and *sul1* genes, forming a highly stable, low cost structure with reported prevalence rates in *E. coli* of 63% (Lacotte et al., 2017; Fang et al., 2019). In addition, in the present study, the *sul2* gene was commonly found to be part of a cassette structure linked with the streptomycin resistance genes *strA* and *strB* in the plasmid of *E. coli* isolates from *P. vannamei*, a feature that has been reported in many other studies in both *E. coli* and *Salmonella* (Enne et al., 2004; Bean et al., 2009; Lopes et al., 2015; Ma̧ka et al., 2015). This cassette is not recognized as an MGE in its own right but has been associated with other MGEs, such as IS*1294*, IS*1131*, and IS*1S*. More importantly, co-location of resistance genes in the same plasmid or fragment, such as genes adjacent to *sul1* found to encode for quinolone resistance (*qnrB4*) or β-lactamase resistance (*blaTEM-1* and *blaDHA-1*) and genes adjacent to *sul2* found to encode for tetracycline resistance (*tetA* and *tetR*), aminoglycoside resistance (*aph* and *aad*) and amphenicol resistance (*floR*), likely contribute to the persistence and dissemination of *sul1* and/or *sul2* genes under the selective pressure of other commonly used antibiotics. This co-selection effect has also been reported in other MGEs of class 2 integrons of *E. coli* isolates (Alonso et al., 2018). However, the prevalence of *sul3* is reportedly much lower than that of *sul1* and *sul2* (Byrne-Bailey et al., 2008; Lopes et al., 2015; Ma̧ka et al., 2015), and *sul3* was rarely detected in the *E. coli* isolates from *P. vannamei* and pork samples in our study. In this study, the diversity of MGEs and resistance genes adjacent to *sul3* was much lower than those adjacent to *sul1* and *sul2*, especially among those located in chromosomes, which reduced the transmission potential of the *sul3* gene. However, other studies have found that *sul3*-carrying plasmids are conjugative and associated with class 1 integrons, and can replace the *sul1* gene to form atypical class 1 integrons. This underscores the potential of *sul3* to become more widespread in the future (Wu et al., 2010).

Finally, since *sul* gene-related fragments in *E. coli* isolates from *P. vannamei* and pork samples were linked to various MGEs, which facilitate intracellular and intercellular mobility, in both plasmids and chromosomes, clonal similarities between *E. coli* isolates from *P. vannamei* samples might be coincidental.

In conclusion, *sul* genes were widely distributed in *E. coli* isolates from both *P. vannamei* and pork samples, with *sul1* and *sul2* being more prevalent than *sul3*. Because *sul* genes are located on both plasmids and chromosomes, diverse gene transfer mechanisms can contribute to the efficient spread of these genes to novel species. This was demonstrated by the presence of conjugative and/or mobilizable plasmids of diverse replicon types harboring *sul* genes and the abundant MGEs located in the flanking regions of these genes. In addition, co-location of resistance genes likely contributed to the persistence and dissemination of *sul* genes under the selective pressure of commonly used antibiotics. Our results provide important information to gain a better understanding of the risk of transmission of *sul* genes from seafood and meat to humans.

## Data Availability

The raw data supporting the conclusions of this manuscript will be made available by the authors, without undue reservation, to any qualified researcher.

## Author Contributions

HJ completed the clonal analysis by molecular typing, location and transferability of *sul-*genes, and replicon typing of plasmids, and prepared the manuscript. HC completed the genetic context analysis of *sul*-genes and prepared the figures and tables. YL, SY, and TY completed the shrimp and pork sampling, *E. coli* isolation, and the phenotypic screening of sulfonamide resistance and detection of *sul*-genes. JF and CZ designed the project, completed the data analysis, and revised the manuscript.

## Conflict of Interest Statement

The authors declare that the research was conducted in the absence of any commercial or financial relationships that could be construed as a potential conflict of interest.

## Footnotes

1 http://www.fao.org/fishery/statistics/global-aquaculture-production/query/en?tdsourcetag=s_pctim_aiomsg/en

2 http://www.moa.gov.cn/govpublic/SYJ/201509/t20150907_4819267.htm

3 http://enterobase.warwick.ac.uk/species/ecoli/allele_st_search

4 https://blast.ncbi.nlm.nih.gov/Blast.cgi

5 http://www-is.biotoul.fr/

## Supplementary Material

FIGURE S1 Detection of *sul* genes in sulfisoxazole-resistant *E. coli* isolates (partial) by PCR. **(A)** detection of *sul1*; **(B)** detection of *sul2*; **(C)** detection of *sul3*. M, DL2000 marker (100, 250, 500, 750, 1000, and 2000 bp). P, positive control; N, negative control; 1−21, sulfisoxazole-resistant *E. coli* isolates.

FIGURE S2 Southern blot analysis of the locations of *sul* genes in *E. coli* isolates. **(A)** Analysis of the location of *sul1*. 1, chromosomal DNA of 1PO-01 isolate; 2, plasmid DNA of 1PO-01 isolate; 3, chromosomal DNA of 2PO -57 isolate; 4, plasmid DNA of 2PO-57 isolate; 5, chromosomal DNA of 3PO-15 isolate; 6, plasmid DNA of 3PO-15 isolate; 7, chromosomal DNA of 1PO-20 isolate; 8, plasmid DNA of 1PO-20 isolate. **(B)** Analysis of the location of *sul2*. 1, plasmid DNA of 1PV-01 isolate; 2, plasmid DNA of 2PV-19 isolate; 3, plasmid DNA of 3PV-52 isolate; 4, plasmid DNA of 1PV-38 isolate; 5, plasmid DNA of 2PV-22 isolate; 6, plasmid DNA of 3PV-15 isolate; 7, plasmid DNA of 3PV-24 isolate. **(C)** Analysis of the location of *sul3*. 1, chromosomal DNA of 1PO-58 isolate; 2, chromosomal DNA of 1PO-27 isolate; 3, chromosomal DNA of 2PO- 11 isolate; 4, chromosomal DNA of 3PV-24 isolate; 5, chromosomal DNA of 2PV-02 isolate; 6, chromosomal DNA of 1PV-44 isolate. M, DNA molecular weight marker II, digoxigenin-labeled (Roche, Penzberg, Germany) with the following base pair lengths: 125, 564, 2027, 2322, 4361, 6557, 9416, and 23,130 bp.

FIGURE S3 Primary PCR products of *sul* gene flanking sequences obtained using the PCR walking method. **(A)** Products of *sul1* gene flanking sequences. **(B)** Products of *sul2* gene flanking sequences. **(C)** Products of *sul3* gene flanking sequences. M: DL5000 marker (100, 250, 500, 750, 1000, 2000, 3000, and 5000 bp). 1−7, products of *sul* gene upstream sequences (DNA template is *Dra*I library, *Eco*RV library, *Hpa*I library, *Pvu*II library, *Sma*I library, *Stu*I library, and *Ssp*I library). 8−14, products of *sul* gene downstream sequences (DNA template is *Dra*I library, *Eco*RV library, *Hpa*I library, *Pvu*II library, *Sma*I library, *Stu*I library, and *Ssp*I library).
