# Corrigendum: Diverse Mobile Genetic Elements and Conjugal Transferability of Sulfonamide Resistance Genes (*sul1, sul2, and sul3*) in *Escherichia coli* Isolates From *Penaeus vannamei* and Pork From Large Markets in Zhejiang, China

**DOI:** 10.3389/fmicb.2020.01793

**Published:** 2020-08-17

**Authors:** Han Jiang, Hui Cheng, Yi Liang, Shengtao Yu, Ting Yu, Jiehong Fang, Cheng Zhu

**Affiliations:** Key Laboratory of Marine Food Quality and Hazard Controlling Technology of Zhejiang Province, College of Life Sciences, China Jiliang University, Hangzhou, China

**Keywords:** sulfonamide resistance genes, *Escherichia coli*, mobile genetic elements, insertion sequences, conjugation

In the original article, there was a mistake in Figure 2C as published. Figure 2C and Figure 2B were repeated. The corrected Figure 2 appears below.

**Figure 2:**
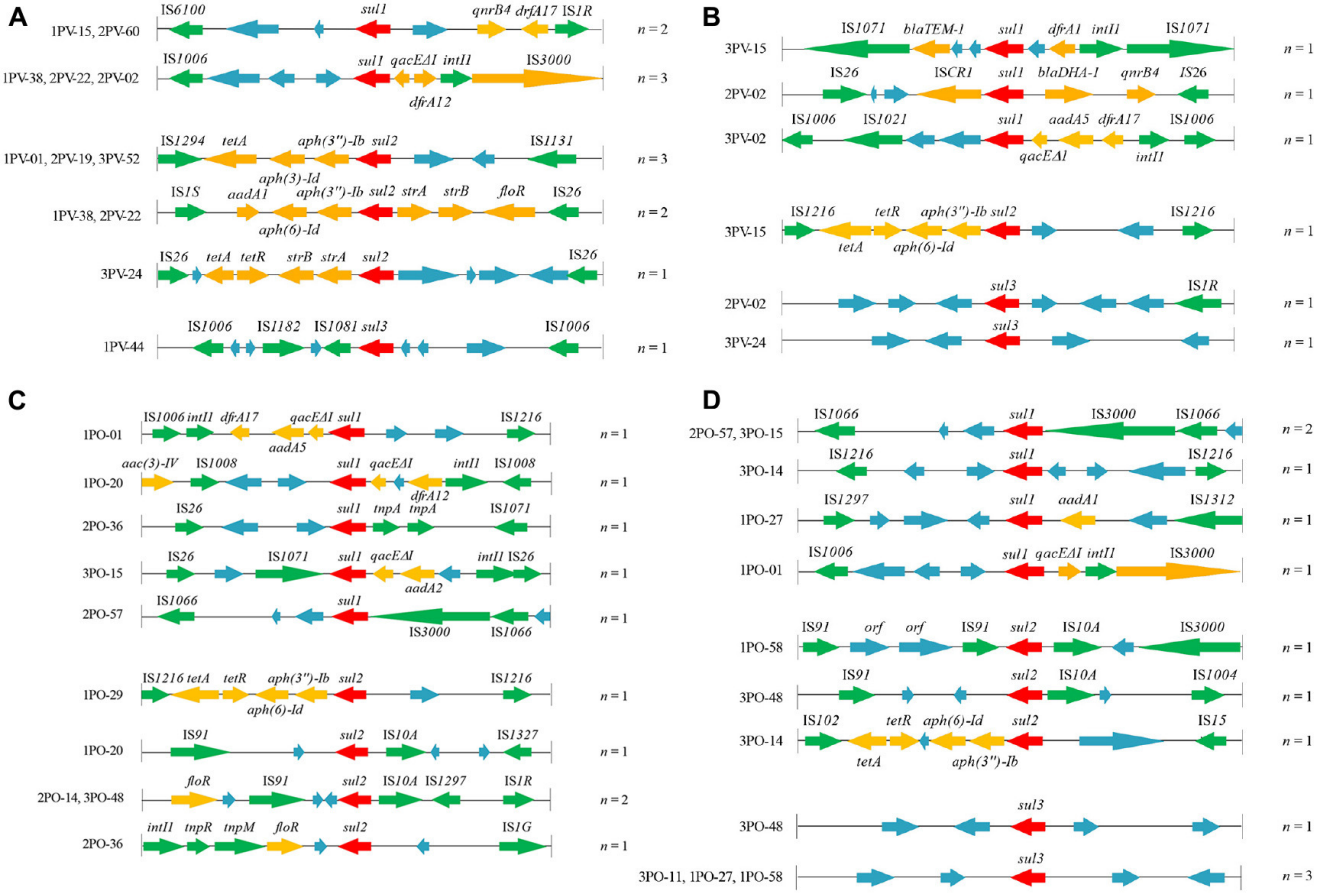
Genetic organization of *sul* gene-associated regions in **(A)** plasmids of 12 *sul*-positive *Escherichia coli* isolates from *Penaeus vannamei*; **(B)** chromosomes of 12 *sul*-positive *E. coli* isolates from *P. vannamei*; **(C)** plasmids of 12 *sul*-positive *E. coli* isolates from pork products; and **(D)** chromosomes of 12 *sul*-positive *E. coli* isolates from pork products presented with their isolate numbers. The orientation of each gene and insertion element is indicated by arrows. The same units are shown in the same color. The same functional units or unknown functional units are shown in the same color (red, *sul* genes; yellow, antibiotic resistance genes other than *sul* genes; green, mobile genetic elements; blue, unknown functional unit). Names of sequence units are indicated above or below the arrows, and sequence units with unknown functions have been left blank.

The authors apologize for this error and state that this does not change the scientific conclusions of the article in any way. The original article has been updated.

